# A Prognostic Nutritional Scoring System Integrating Inflammatory Markers and Body Composition in Gastric Cancer Patients Undergoing Curative Resection With Prophylactic HIPEC

**DOI:** 10.1155/grp/7959410

**Published:** 2026-05-14

**Authors:** Ruiqing Liu, Zhibo Wang, Jingnong Liu, Yahya Ahmed, Yuandi Wang, Dongsheng Wang, Duo Li

**Affiliations:** ^1^ Department of Gastrointestinal Surgery, The Affiliated Hospital of Qingdao University, Qingdao, China, qdu.edu.cn; ^2^ Institute of Nutrition and Health, College of Public Health, Qingdao University, Qingdao, China, qdu.edu.cn; ^3^ Department of General Surgery, Weifang People’s Hospital, Weifang, China, wfph.cn; ^4^ Division of Surgical Oncology, Mayo Clinic Florida, Jacksonville, Florida, USA, mayoclinic.org

**Keywords:** body composition, gastric cancer, inflammatory nutrition markers, prophylactic intraperitoneal hyperthermic chemotherapy

## Abstract

**Background and Objectives:**

Nutrition‐ and inflammation‐related indicators have been shown the prognostic value in cancer patients; however, few studies focus on gastric cancer (GC) patients who underwent prophylactic intraperitoneal hyperthermic chemotherapy (p‐HIPEC) following radical gastrectomy. This study is aimed at developing and validating a prognostic nutritional scoring system (PNSS) integrating systemic inflammatory markers and body composition parameters specifically for these patients.

**Methods and Results:**

Body composition was assessed via computed tomography, and systemic inflammatory markers were recorded preoperatively. A total of 267 patients with GC who received p‐HIPEC were included, and the incidences of sarcopenia, myosteatosis, and sarcopenic obesity were 32.6%, 27.3%, and 18.7%, respectively. Logistic regression analysis identified that platelet‐to‐lymphocyte ratio (PLR) is a significant risk factor for sarcopenia and myosteatosis. The PNSS was constructed using PLR, systemic immune‐inflammation index (SII), lymphocyte‐to‐monocyte ratio (LMR), skeletal muscle index (SMI), and sarcopenia‐myosteatosis risk (SMR) score which further integrates skeletal muscle attenuation (MA). This model demonstrated superior predictive accuracy for overall survival (OS) compared to the classic TNM model (*C*‐index: 0.76 vs. 0.71 for 3‐year OS in the primary cohort and 0.68 vs. 0.64 for 5‐year OS in the validation cohort).

**Conclusions:**

The PNSS, incorporating both body composition and systemic inflammatory markers, provides robust prognostic value for predicting OS in GC patients undergoing curative resection with p‐HIPEC.

## 1. Introduction

Gastric cancer is the fifth most common malignancy and the third leading cause of cancer‐related morbidity worldwide [1]. Radical gastrectomy with lymphadenectomy and adjuvant chemotherapy remains the cornerstone of curative treatment for localized advanced gastric cancer, whereas approximately 30%–50% of patients experience recurrence after radical resection, especially for those with clinical T4 gastric cancer [2]. Peritoneal metastasis (PM) is the most common and earliest form of recurrence in GC following radical surgery, associated with a 5‐year survival rate for less than 30%, significantly lower in GC without PM [3]. It is challenging to detect PM during routine follow‐up, and the effect of typical systemic chemotherapy is limited [4].

To combat this, intraperitoneal hyperthermic chemotherapy (HIPEC) has been explored as a promising treatment. HIPEC delivers heated chemotherapy directly into the peritoneal cavity after surgery, targeting microscopic disease undetectable during surgery. The combination of hyperthermia and chemotherapy enhances drug penetration, induces tumor cell apoptosis, and reduces peritoneal recurrence [5, 6]. Systemic reviews suggest that p‐HIPEC can be performed safely in locally advanced gastric cancer (LAGC), preventing PM and potentially improving survival [7, 8]. A recent randomized controlled trial by Yu et al. showed significantly lower PM incidence in the p‐HIPEC group (20.9% vs. 40.3%, *p* = 0.015), with comparable adverse event rates, supporting its safety and feasibility [9]. Despite these findings, p‐HIPEC is not yet recommended by the National Comprehensive Cancer Network (NCCN), as its survival benefits in broader LAGC populations have not been definitively established [10]. This variability in outcomes underscores the need for careful preoperative evaluation of LAGC patients for p‐HIPEC, particularly to identify factors influencing treatment failure.

Malnutrition is a critical prognostic factor in GC, depleting energy reserves, weakening immune function, and impairing the body’s ability to combat disease [11, 12]. To date, many objective nutritional‐related indicators are recognized to have a great prognostic value for GC patients, among which, body composition has emerged as a critical prognostic factor [13]. Abnormal body composition parameters indicative of poor conditions, such as sarcopenia, myosteatosis (the pathological infiltration of fat into skeletal muscle [SM]), visceral obesity, and sarcopenic obesity (the coexistence of reduced muscle mass and excess visceral adiposity), which are strongly associated with increased postoperative complications, prolonged recovery, and reduced overall survival (OS) in GC patients [14, 15]. Inflammation is also a significant factor in cancer progression [16]. Systemic inflammatory indicators like neutrophil‐to‐lymphocyte ratio (NLR), PLR, platelet‐to‐albumin ratio (PAR), prognostic nutritional index (PNI), SII, and LMR provide valuable insight into the inflammatory burden, which is closely tied to nutritional status [17, 18]. Inflammation disrupts metabolic balance, leading to muscle wasting, fat depletion, and overall nutritional decline. Thus, preoperative screening that integrates both body composition and inflammatory status is vital for assessing HIPEC treatment eligibility in GC patients.

Given these perspectives, this study is aimed at quantifying body composition and nutritional inflammatory indicators in LAGC patients undergoing radical surgery followed by p‐HIPEC and at exploring the association between these objective parameters and oncological outcomes. Ultimately, it is also aimed at developing a prognostic scoring system to integrate these parameters to predict OS in this population.

## 2. Materials and Methods

### 2.1. Patient Selection

**Figure 1:**
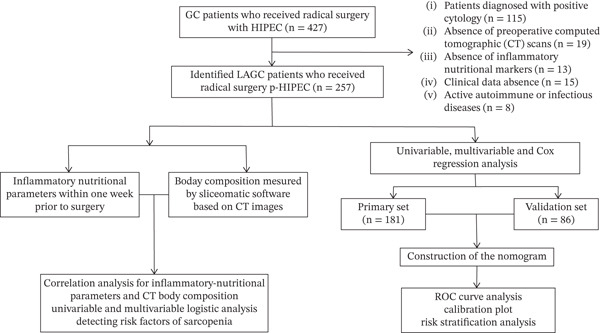
Flow chart for the inclusion and exclusion of localized advanced gastric cancer patients and construction of prognostic nutritional scoring system.

This retrospective study included 267 GC patients who underwent radical gastrectomy and prophylactic HIPEC at two campuses of our institution. The primary cohort consisted of 181 patients from Campus 1 (January 2017–December 2021), and the validation cohort comprised 86 patients from Campus 2 (March 2018–April 2022). The study was approved by the local Ethics Committee and adhered to the Declaration of Helsinki. A flow diagram outlining the study design is presented in Figure 1. Clinicopathological data, including age, gender, American Society of Anesthesiologists (ASA) score, oncological characteristics, and treatment details, were extracted from electronic records.

### 2.2. Inclusion and Exclusion Criteria

The inclusion criteria are as follows: patients aged between 18 and 75 years; histologically confirmed diagnosis of gastric adenocarcinoma; clinical Stage T4 based on radiologic evaluation, with or without lymph node metastasis; and no evidence of distant metastasis, including liver, lung, or PM.

The exclusion criteria are as follows: positive cytology from laparoscopy, active inflammatory conditions requiring systemic immunosuppressive therapy, concurrent malignancy, and absence of preoperative computed tomography (CT) scans or inflammatory–nutritional markers.

### 2.3. Evaluation of Inflammatory and Nutritional Parameters

**Figure 2:**
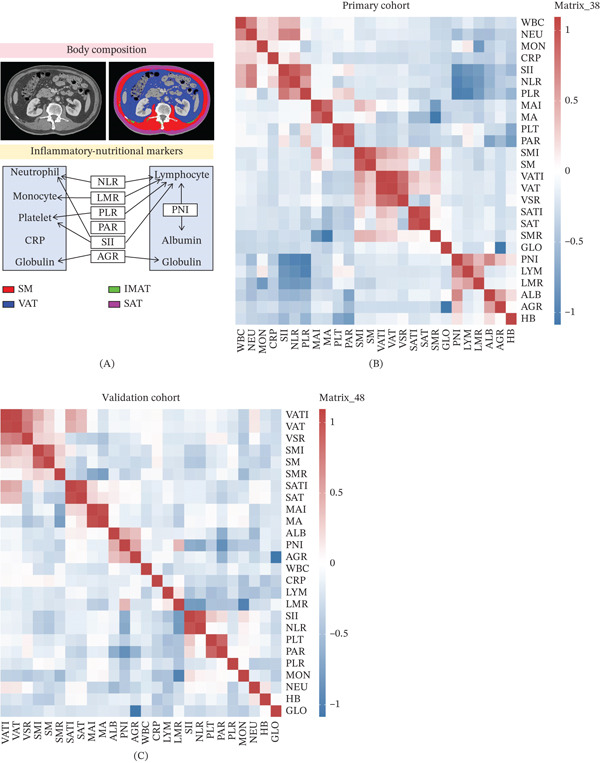
The process of (A) generating and performing correlation analysis for body composition and inflammatory–nutritional markers: (B) primary cohort and (C) validation cohort.

Blood samples were collected within 2 weeks before surgery. Evaluated laboratory parameters included neutrophil, platelet, lymphocyte, monocyte, hemoglobin, albumin (ALB), white blood cell count (WBC), C‐reactive protein (CRP), and globulin (GLO). The following combined markers were calculated: NLR, PLR, PAR, PNI, SII, LMR, and albumin‐to‐globulin ratio (AGR) (Table S1 and Figure 2A).

### 2.4. Body Composition Assessment

Body composition was assessed using CT scans at the L3 vertebral level taken within 1 month prior to surgery. Measurements included SM, visceral adipose tissue (VAT), subcutaneous adipose tissue (SAT), MA, and SMR score which further integrates SM attenuation. Image analysis was performed using SliceOmatic software, with cross‐sectional areas of SM, VAT, and SAT quantified based on Hounsfield unit (HU) thresholds. SM area was defined within the attenuation range of −29 to +150 HU, VAT as −150 to −50 HU, and SAT as −190 to −30 HU, consistent with previously established protocols [19]. Body composition measurements were normalized for height to derive the SMI, visceral adipose index (VAI), and subcutaneous adipose index (SAI), as well as the visceral adipose‐to‐subcutaneous adipose tissue ratio (VSR) (Figure 2B). Sarcopenia was diagnosed using sex‐ and BMI‐specific body composition thresholds (Table S1) [14, 15, 20].

### 2.5. Treatment Method

All patients underwent standardized radical gastrectomy with D2 lymphadenectomy. Prophylactic HIPEC was administered to patients with T4 GC, based on preoperative imaging and intraoperative findings. After gastrectomy and wound closure, an inflow catheter was positioned in the small omental sac, and three outflow catheters were placed in the pelvis and subhepatic area. The HIPEC regimen consisted of either paclitaxel (120 mg) or raltitrexed (4 mg), dissolved in 3000 mL of saline, perfused for 60 min at 42°C ± 1°C. Following HIPEC, patients received adjuvant chemotherapy (SOX or XELOX), with regimen adjustments based on disease progression or side effects.

### 2.6. Follow‐Up

Patients were followed regularly, with assessments every 3 months during the first 2 years and every 6 months thereafter. Follow‐up included physical exams, tumor markers, and imaging. OS was defined as the time from surgery to death or last follow‐up.

### 2.7. Construction of the PNSS

Univariate and multivariate Cox regression analyses identified independent prognostic factors, with results presented as odds ratios (ORs) and 95% confidence intervals (CIs). A prognostic scoring system was developed based on preoperative factors with *p* values < 0.1. A nomogram was created, and the predictive accuracy of the PNSS for OS was evaluated using the area under the receiver‐operating characteristic (ROC) curve (AUC). The performance of the nomogram was assessed using both the primary and validation cohorts, with calibration curves generated for comparison of predicted and actual survival outcomes. Cutoff values for indicators were derived using X‐tile software [21].

### 2.8. Statistical Analysis

Categorical variables were analyzed using chi‐square or Fisher’s exact tests, and continuous variables were analyzed using the Student’s *t*‐test. Survival curves were estimated using the Kaplan–Meier (K‐M) methods, with differences assessed by the log‐rank test. Patients with missing data for any of the key inflammatory–nutritional markers or body composition parameters were excluded from the analysis to ensure data integrity. Statistical analyses were conducted using SPSS 24 and R statistical software, with a *p* value < 0.05 considered statistically significant.

## 3. Results

### 3.1. Patient Characteristics

A total of 267 patients were included in the study, with 181 in the primary cohort and 86 in the validation cohort (Table 1). Among all patients, 66.3% were male, and 61.8% were aged ≥ 60 years. Approximately 68.5% of patients were at nutritional risk, with an NRS‐2002 score > 2. Pathological staging revealed that 60.7% of patients had pT4 tumors and 41.2% had pN3 lymph node involvement. Prior to surgery, 10.9% received neoadjuvant chemotherapy. Table 2 compares the body composition and systemic inflammatory parameters between the groups. The percentages of sarcopenia, myosteatosis, visceral obesity, and sarcopenic obesity were 32.6%, 27.3%, 41.6%, and 18.7%, respectively. A slight but marginally significant difference in SMR was found between cohorts (*p* = 0.05).

**Table 1:** Baseline patient characteristics.

	Overall patients (*n* = 267)	Primary cohort (*n* = 181)	Validation cohort (*n* = 86)	*p* value
Gender, *n* (%)				0.779
Male	177 (66.3)	121 (66.9)	56 (65.1)	
Female	90 (33.7)	62 (33.1)	30 (34.9)	
Age (years), *n* (%)				0.264
≥ 60	165 (61.8)	116 (64.1)	49 (57.0)	
< 60	102 (38.2)	65 (35.9)	37 (43.0)	
BMI (kg/m^2^), *n* (%)				0.159
> 18.5	13 (4.9)	6 (3.3)	7 (8.1)	
≤ 18.5	254 (95.1)	175 (96.7)	79 (91.9)	
Comorbidity, *n* (%)				0.892
Yes	135 (50.6)	91 (50.3)	44 (51.2)	
No	132 (49.4)	90 (49.7)	42 (48.8)	
NRS‐2002, *n* (%)				0.507
> 2	183 (68.5)	125 (69.1)	58 (67.4)	
≤ 2	84 (31.5)	56 (30.9)	28 (32.6)	
ASA, *n* (%)				0.788
> 2	126 (47.2)	72 (39.8)	32 (37.2)	
≤ 2	141 (52.8)	109 (60.2)	54 (62.8)	
Tumor site, *n* (%)				0.204
Cardia and body	120 (44.9)	85 (47.0)	35 (40.7)	
Antrum	147 (55.1)	96 (53.0)	51 (59.3)	
Histology, *n* (%)				0.594
Nonadenocarcinoma	39 (14.6)	25 (13.8)	14 (16.3)	
Adenocarcinoma	228 (85.4)	156 (86.2)	72 (83.7)	
Tumor size (cm), *n* (%)				0.286
> 5	143 (53.6)	101 (55.8)	42 (48.8)	
≤ 5	124 (46.4)	80 (44.2)	44 (51.2)	
pT stage, *n* (%)				0.584
T1–T3	105 (39.3)	62 (34.3)	33 (38.4)	
T4	162 (60.7)	119 (65.7)	53 (61.6)	
pN stage, *n* (%)				0.703
N0–N2	157 (58.8)	105 (58.0)	52 (60.5)	
N3	110 (41.2)	76 (42.0)	34 (39.5)	
Differentiation degree, *n* (%)				0.526
Well or moderately	74 (27.7)	48 (26.5)	26 (30.2)	
Poor	193 (72.3)	133 (73.5)	60 (69.8)	
Vascular invasion, *n* (%)				0.849
Yes	176 (65.9)	120 (66.3)	56 (65.1)	
No	91 (34.1)	61 (33.7)	30 (34.9)	
Neoadjuvant therapy, *n* (%)				0.573
Yes	29 (10.9)	21 (11.6)	8 (9.3)	
No	238 (89.1)	160 (88.4)	78 (90.7)	

Abbreviations: BMI = body mass index, NRS‐2002 = Nutritional Risk Screening 2002, pN = pathological N, pT = pathological T.

**Table 2:** Assessment of inflammatory–nutritional markers and body compositions.

Variable	Total cohort (*n* = 267)	Primary cohort (*n* = 181)	Validation cohort (*n* = 86)	*p* value
Laboratory parameters				
NEU (10^9^/L)	3.75 (2.93–4.82)	3.76 (2.98–5.01)	3.54 (2.81–4.67)	0.206
PLT (10^9^/L)	284.00 (239.00–340.00)	284.00 (236.50–342.50)	278.00 (240.00–331.25)	0.236
LYM (10^9^/L)	1.82 (1.30–2.45)	1.81 (1.31–2.43)	1.83 (1.27–2.78)	0.27
ALB (g/L)	38.00 (34.84–40.85)	38.50 (35.47–40.87)	37.31 (34.05–40.84)	0.142
MON (10^9^/L)	0.43 (0.35–0.61)	0.43 (0.35–0.62)	0.44 (0.36–0.61)	0.777
HB (g/L)	123.00 (102.00–139.00)	122.00 (103.00–138.00)	126.00 (101.00–139.00)	0.68
WBC (10^9^/L)	6.16 (5.01–7.20)	6.21 (5.03–7.41)	6.11 (4.81–7.04)	0.067
CRP (mg/L)	2.50 (1.50–4.40)	2.76 (1.57–4.05)	2.08 (1.40–4.53)	0.205
GLO (g/L)	25.84 (23.30–28.50)	25.57 (23.22–28.01)	26.67 (23.45–29.16)	0.156
Inflammatory–nutritional markers				
NLR	2.16 (1.48–3.25)	2.03 (1.36–3.01)	2.47 (1.79–3.83)	0.139
PLR	158.28 (110.43–226.82)	158.10 (105.47–225.19)	164.51 (114.47–263.51)	0.188
PAR	7.73 (6.20–9.42)	7.73 (6.11–9.63)	7.76 (6.43–9.26)	0.779
PNI	48.63 (43.49–52.06)	48.65 (43.53–51.95)	48.30 (43.41–52.60)	0.838
SII	600.00 (400.83–989.25)	568.20 (375.01–967.28)	676.66 (456.44–1113.59)	0.138
LMR	3.92 (2.83–5.46)	3.95 (2.78–5.46)	3.85 (2.88–5.41)	0.899
AGR	1.49 (1.31–1.64)	1.49 (1.31–1.64)	1.48 (1.31–1.65)	0.521
Body composition markers				
SM (cm^2^)	131.60 (110.90–150.00)	129.50 (108.95–150.10)	134.55 (115.80–149.72)	0.109
VAT (cm^2^)	98.48 (47.04–146.90)	97.73 (46.95–141.90)	101.55 (46.15–154.67)	0.467
SAT (cm^2^)	119.10 (83.99–151.20)	120.00 (89.22–150.35)	113.10 (70.07–152.80)	0.43
MA (cm^2^)	41.74 (30.79–47.08)	40.90 (33.07–44.91)	44.42 (24.20–53.38)	0.657
SMI (cm^2^/m^2^)	44.21 (35.71–54.27)	44.75 (35.18–54.06)	43.59 (37.66–55.09)	0.705
VSR (VAT/SAT)	0.78 (0.48–1.20)	0.73 (0.43–1.21)	0.87 (0.52–1.14)	0.199
VATI (cm^2^/m^2^)	32.71 (14.32–53.14)	32.74 (14.68–52.45)	31.66 (13.38–55.48)	0.647
SATI (cm^2^/m^2^)	40.73 (27.83–53.32)	41.76 (29.46–53.00)	35.56 (23.18–55.18)	0.289
SMR (SM ∗ MA)	3.11 (2.51–3.67)	3.29 (2.85–3.81)	3.01 (1.97–3.58)	0.05
MAI (HU)	13.85 (10.55–16.26)	13.84 (11.69–15.99)	13.87 (7.53–17.28)	0.17
Sarcopenia‐related diagnosis				
Sarcopenia	87 (32.6)	63 (34.8)	24 (27.9)	0.328
Myosteatosis	73 (27.3)	43 (23.8)	30 (34.9)	0.08
Visceral obesity	111 (41.6)	80 (44.2)	31 (36.0)	0.23
Sarcopenia obesity	50 (18.7)	36 (19.9)	14 (16.3)	0.51

*Note:* Continuous parameters are presented as median (interquartile ranges Q1–Q3), and categorical parameters are presented as *n* (%).

Abbreviations: AGR = albumin‐to‐globulin ratio, ALB = albumin, CRP = C‐reactive protein, GLO = globulin, HB = hemoglobin, HU = Hounsfield unit, IQR = interquartile range, LMR = lymphocyte‐to‐monocyte ratio, LYM = lymphocytes, MA = muscle attenuation (a measure of muscle density/fat content), MAI = muscle attenuation index, MON = monocyte, NEU = neutrophil, NLR = neutrophil‐to‐lymphocyte ratio, PAR = platelet‐to‐albumin ratio, PLR = platelet‐to‐lymphocyte ratio, PLT = platelet, PNI = prognostic nutritional index, SAT = subcutaneous adipose tissue area, SATI = subcutaneous adipose tissue index (SAT normalized for height), SII = systemic immune‐inflammation index, SM = skeletal muscle area, SMI = skeletal muscle index (SM normalized for height), SMR = sarcopenia‐myosteatosis risk score, VAT = visceral adipose tissue area, VATI = visceral adipose tissue index (VAT normalized for height), VSR = visceral‐to‐subcutaneous fat ratio, WBC = white blood cells.

### 3.2. Correlations Between Body Composition and Inflammatory Markers

Systemic inflammatory markers and body composition data were collected. The Pearson correlation analysis revealed no strong linear correlations (|*r*| > 0.75) between body composition and inflammatory markers, suggesting that these could be combined into a multivariable model (Figure 2B,C and Table S2). Analysis of survival data using X‐tile determined cutoff values for body composition and inflammation parameters that significantly differentiated low‐ and high‐risk groups. These included NLR, PLR, LMR, SMI, MA, subcutaneous adipose tissue index (SATI), VSR, and SMR (log‐rank *p* < 0.05, Figure S1). Univariable and multivariable logistic regression analyses revealed that male gender (OR 4.285, *p* < 0.001), age ≥ 60 years (OR 0.255, *p* < 0.001), and PLR (OR 0.26, *p* < 0.001) were significantly associated with sarcopenia. For myosteatosis, age ≥ 60 years (OR 0.24, *p* < 0.001) and PLR (OR 3.198, *p* = 0.002) were significant predictors (Table 3).

**Table 3:** Univariate and multivariate logistic analysis among clinicopathologic parameters, systemic inflammatory markers, and poorer body composition conditions.

Variables	Sarcopenia						Myosteatosis					
	Univariable			Multivariable			Univariable			Multivariable		
	OR	95% CI	*p* value	OR	95% CI	*p* value	OR	95% CI	*p* value	OR	95% CI	*p* value
Gender, male	0.37	0.19–0.717	0.003	4.285	2.048–8.965	< 0.001	0.726	0.342–1.541	0.404			
Age, ≥ 60	3.318	1.73–6.363	< 0.001	0.255	0.121–0.538	< 0.001	0.235	0.102–0.544	0.001	0.241	0.103–0.568	0.001
BMI	0.254	0.045–1.429	0.12				0.752	0.272–2.076	0.582			
T stage	0.939	0.492–1.792	0.849				0.501	0.228–1.101	0.085			
N stage	1.288	0.695–2.389	0.421				1.479	0.726–3.012	0.281			
Histology	0.681	0.365–1.271	0.228				4.1	0.926–18.16	0.063			
Tumor size	1.823	0.688–4.829	0.227				0.602	0.296–1.225	0.161			
Differentiation degree	1.325	0.67–2.621	0.418				1.097	0.509–2.362	0.813			
NRS‐2002 (> 2)	0.754	0.392–1.45	0.398				1.895	0.93–3.861	0.078			
NLR (> 1.9)	1.046	0.566–1.933	0.886				1.066	0.536–2.118	0.855			
PLR (> 128.7)	0.307	0.157–0.602	0.001	0.26	0.125–0.54	< 0.001	3.285	1.604–6.728	0.001	3.198	1.524–6.712	0.002
PAR (< 5.0)	0.797	0.309–2.052	0.638				1.483	0.566–3.883	0.423			
PNI (< 49.5)	1.083	0.586–2.001	0.8				1.241	0.625–2.465	0.538			
SII (< 423.4)	0.694	0.354–1.361	0.288				1.547	0.759–3.153	0.23			
LMR (> 4.7)	0.881	0.41–1.896	0.747				0.73	0.318–1.674	0.457			

Abbreviations: AGR = albumin‐to‐globulin ratio, BMI = body mass index, CI = confidence interval, IQR = interquartile range, LMR = lymphocyte‐to‐monocyte ratio, NLR = neutrophil‐to‐lymphocyte ratio, NRS‐2002 = Nutritional Risk Screening 2002, OR = odds ratio, PAR = platelet‐to‐albumin ratio, PLR = platelet‐to‐lymphocyte ratio, PNI = prognostic nutritional index, SII = systemic immune‐inflammation index.

### 3.3. Survival Analysis

The median follow‐up time was 40.07 months (6.5–74.6 months) in the primary cohort, with 3‐ and 5‐year OS rates of 63.3% and 39.1%, respectively. In the validation cohort, the median follow‐up was 37.58 months (6.3–75.7 months), with 3‐ and 5‐year OS rates of 70.7% and 52.2%. Cox regression analyses revealed that the pT, PLR, LMR, and SMI were independent prognostic factors for OS in the primary cohort (Table 4). In the validation cohort, PLR and SMR remained significant prognostic factors (Table S3).

**Table 4:** Univariate and multivariate Cox analysis in training cohort among clinicopathologic parameters, systemic inflammatory markers, and poorer body composition conditions.

	Univariable			Multivariable		
	HR	95% CI	*p* value	HR	95% CI	*p* value
Age	0.67	0.43–1.05	0.08			
Gender	0.92	0.6–1.41	0.70			
T stage	1.89	1.19–2.88	0.01	1.584	0.98–2.55	0.06
N stage	2.24	1.49–3.37	0.00	2.533	1.63–3.93	< 0.001
NRS‐2002 (> 2)	1.04	0.67–1.62	0.85			
Histology	0.66	0.34–1.27	0.21			
Tumor size	1.29	0.85–1.95	0.23			
Differentiation degree	1.32	0.81–2.12	0.26			
Vascular invasion	1.38	0.89–2.16	0.15			
Neoadjuvant therapy	1.52	0.86–2.65	0.14			
NLR (> 1.9)	1.61	1.06–2.43	0.03	0.709	0.403–1.25	0.233
PLR (> 128.7)	2.30	1.46–3.62	0.00	1.813	1.08–3.04	0.024
PAR (< 5.0)	1.80	0.87–3.73	0.11			
PNI (< 49.5)	1.46	0.96–2.20	0.08			
SII (< 423.4)	2.03	1.25–3.27	0.00	1.757	0.90–3.43	0.099
LMR (> 4.7)	0.39	0.20–0.76	0.01	0.432	0.22–0.86	0.018
SMI (< 40.1)	1.96	1.29–2.94	0.00	1.756	1.07–2.88	0.025
MA (< 44.6)	1.84	1.21–2.79	0.00	1.276	0.77–2.11	0.343
VSR (< 1.6)	1.44	0.95–2.15	0.08			
VATI (< 24.0)	1.41	0.93–2.13	0.10			
SATI (> 47.5)	1.56	1.00–2.42	0.05	1.384	0.86–2.22	0.178
SMR (< 2.9)	2.64	1.74–4.00	0.00	1.685	0.96–2.96	0.07
MAI (< 10.6)	1.22	0.73–2.04	0.46			

Abbreviations: AGR = albumin‐to‐globulin ratio, BMI = body mass index, CI = confidence interval, HR = hazard ratio, IQR = interquartile range, LMR = lymphocyte‐to‐monocyte ratio, MA = muscle attenuation (a measure of muscle density/fat content), MAI = muscle attenuation index, NLR = neutrophil‐to‐lymphocyte ratio, NRS‐2002 = Nutritional Risk Screening 2002, PAR = platelet‐to‐albumin ratio, PLR = platelet‐to‐lymphocyte ratio, PNI = prognostic nutritional index, SAT = subcutaneous adipose tissue area, SATI = subcutaneous adipose tissue index (SAT normalized for height), SII = systemic immune‐inflammation index, SM = skeletal muscle area, SMI = skeletal muscle index (SM normalized for height), SMR = sarcopenia‐myosteatosis risk score, VAT = visceral adipose tissue area, VAT = visceral adipose tissue index (VAT normalized for height), VSR = visceral‐to‐subcutaneous fat ratio.

### 3.4. Construction and Validation of the PNSS

**Figure 3:**
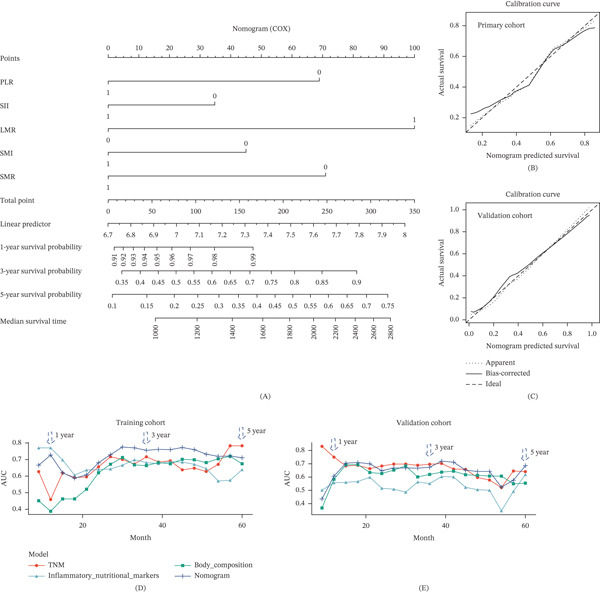
Nomograms for PNSS model development and calibration. (A) A nomogram to predict the 1‐, 3‐, and 5‐year OS of LAGC patients with HIPEC. Calibration plot in the (B) primary and (C) validation cohort. Comparison of the predictive capabilities between the current prognostic model and traditional TNM staging in (D) primary cohort and (E) validation cohort.

Using clinicopathological factors (pT and pN), inflammatory–nutritional parameters (PLR, SII, and LMR), and body composition parameters (SMI and SMR), a PNSS (all with *p* < 0.1) was developed. Cox regression analysis was used to assign weights to each parameter, and a nomogram was created (Figure 3A). Calibration plots showed strong alignment with the ideal model, and time‐dependent ROC curves demonstrated that the PNSS outperformed both the inflammatory–nutritional and body composition models in predicting OS (Figure 3B–E). The PNSS also showed superior predictive accuracy compared to the classic TNM model, with higher concordance indices (*C*‐index) for 3‐year OS in the primary cohort (0.71 vs. 0.76) and 5‐year OS in the validation cohort (0.64 vs. 0.68) (Figure 3D,E and Table S4).

### 3.5. Risk Group Stratification Based on PNSS

**Figure 4:**
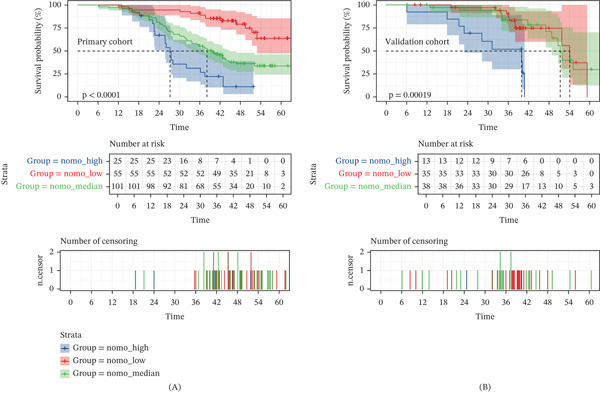
The performance of risk stratification using the prognostic nomogram model in the (A) primary cohort and (B) validation cohort.

Based on the PNSS, patients were categorized into low‐risk, median‐risk, and high‐risk groups. In the primary cohort, the high‐risk group showed significantly poorer OS compared to the low‐risk group (log‐rank *p* < 0.001). Similarly, the validation cohort showed a significant difference in OS between high‐risk and low‐risk groups (log‐rank *p* < 0.001, Figure 4). The survival data for these groups are detailed in Table S5.

## 4. Discussion

This study examined the relationship between body composition, systemic inflammatory markers, and their prognostic significance in GC patients who underwent radical gastrectomy and p‐HIPEC. The findings revealed that PLR was associated with sarcopenia and myosteatosis. Survival analysis identified PLR, LMR, and SMI as independent prognostic factors for OS. The PNSS incorporating systemic inflammation and body compositions demonstrated strong predictive value for OS, highlighting the importance of nutrition‐related factors in this scenario.

Prophylactic HIPEC is an increasingly used therapeutic approach for LAGC patients at high risk of PM. The meta‐analysis by Desiderio et al. showed a significant reduction in overall disease recurrence (relative risk [RR], 0.73; *p* = 0.002) and improved 3‐year (RR 0.71; *p* = 0.03) and 5‐year survival outcomes (RR 0.82; *p* = 0.01) [22]. Similarly, a meta‐analysis by Sun et al. demonstrated notable reductions in both mortality (RR 0.73; *p* < 0.001) and peritoneal recurrence (RR 0.45; *p* = 0.001) in T4a GC patients [23]. Our previous study showed that p‐HIPEC effectively prevented postoperative PM without increasing the incidence of postoperative complications in clinical T4 GC patients [24]. However, HIPEC risks in a prophylactic setting remain controversial owing to the variability in published literature. The combined effects of chemotherapy and hyperthermia also raise concerns about perioperative hypothermia, toxicity, and complications [25].

Candidates for p‐HIPEC are a unique subset of LAGC patients, as they face risks for PM, and the unpredictable treatment effects of HIPEC. The HIPEC procedure following radical gastrectomy, administered in 2–3 cycles postoperatively under repeated anesthesia, can lead to a complex stress response, food restriction, and gastrointestinal dysfunction. Common side effects associated with p‐HIPEC include hypermetabolic state, fluid and electrolyte imbalances, renal failure, systemic inflammatory response syndrome (SIRS), and prolonged ileus [9, 26]. Intraperitoneal chemotherapy can increase intestinal permeability, which may reduce nutrient absorption and elevate the risk of systemic infections [27]. These factors collectively affect the patient’s ability to maintain proper nutritional status. Consistent with other evidence [28], our previous study demonstrated that postoperative hypoalbuminemia is more common in patients receiving p‐HIPEC, indicating a higher risk of malnutrition with p‐HIPEC [24]. Perioperative malnutrition is closely associated with prolonged hospital stays, increased chemotherapy and surgical complications, higher readmission rates, and greater mortality in GC patients who underwent HIPEC [28]. Funk‐Debleds et al. reported a 35% increase in the rate of malnutrition during the immediate postsurgery period, while severe malnutrition rose by 83% during the surgical period in GC patients treated with HIPEC. A loss of more than 20% of their usual weight at discharge is associated with a poorer 3‐year OS [12]. A meta‐analysis by Giorgio et al. found that low SM mass at diagnosis is a significant prognostic factor for the development of postoperative complications in metastatic colorectal cancer patients undergoing cytoreductive surgery (CRS) and HIPEC [29]. In the current study, a preoperative sarcopenia rate of 32.6% was observed, slightly higher than that reported in previous studies focused on resectable GC patients. We also found a sarcopenic obesity rate of 18.7% and myosteatosis of 27.3% in the present cohort, reflecting the coexistence of SM deletion and visceral adipose accumulation, which were demonstrated to contribute to shorter survival, increased chemotherapy toxicity, and postoperative complications previously [30, 31]. These findings bring out the increased baseline nutritional risk in HIPEC‐treated patients.

Routine malnutrition screening is therefore recommended for all patients scheduled for resection and HIPEC [28]. It helps identify nutritional prognostic factors and select patients for resection, potentially modifying treatment protocol that could improve oncological outcomes. However, there is currently no consensus on the best nutrition screening or assessment tool to use in the HIPEC context, and several validated tools including the Malnutrition Universal Screening Tool (MUST), Nutritional Risk Screening 2002 (NRS‐2002), and Patient‐Generated Subjective Global Assessment–Short Form (PONS) are relatively subjective and have limited applicability to specific groups [32]. As in this study, no significant correlation was found between NRS‐2002 scores and OS in GC patients treated with p‐HIPEC. Accumulating evidence has demonstrated that both body composition and systemic inflammatory–nutritional markers are potentially useful in predicting clinical outcomes of various cancers [28, 33]. However, most studies have examined these factors separately [14, 28–31], and their combined prognostic value in HIPEC patients remains uncertain. The inflammatory alterations within tumor microenvironment are pivotal in gastric cancer progression [34]. Immune and inflammatory cells are integral to the tumor microenvironment and can promote tumor growth by secreting inflammatory mediators [35]. Chronic inflammation accelerates metabolism and disrupts cellular programming, making cancer patients more vulnerable to malnutrition, ultimately resulting in cachexia if untreated [20]. It is noticeable that HIPEC induces a significant inflammatory response, both locally in the peritoneal cavity and systemically, which might contribute to catabolic metabolism, where the body breaks down muscle and fat for energy. This increases the risk of sarcopenia and body fat loss, both of which are signs of malnutrition. In this study, the associations between body composition, inflammatory–nutritional markers, and their prognostic significance were evaluated in LAGC patients who underwent radical surgery and p‐HIPEC. Our analysis identified a correlation between high PLR and poor body compositions including sarcopenia and myosteatosis, which was in line with the previous clinical findings [36]. Lymphocytopenia indicates that the cellular immune system is unable to function properly, preventing the establishment of an appropriate inflammatory response [37]. In addition, platelet activation induced by systemic inflammation may deteriorate during intraperitoneal chemotherapy, as chemotherapeutic agents like cisplatin or mitomycin C can suppress bone marrow function, potentially leading to thrombocytopenia [23]. In this regard, body composition and inflammatory indices are mechanistically linked, and their combination may provide a comprehensive understanding of the immune‐metabolic condition in LAGC patients who undergo p‐HIPEC.

Body composition and systemic inflammation parameters (SMI, SMR, PLR, SII, and LMR) coincide with recent studies highlighting the significance of sarcopenia and inflammatory response in predicting OS [15, 38, 39]. SMR, which refers to the density of muscle tissue, is a novel indicator used to assess SM quality in clinical settings. SMR adds prognostic value in evaluating the nutritional and functional status of patients with chronic disease [40]. Additionally, it was reported that the potential of PLR, SII, and LMR could serve as postsurgical prognostic markers in GC [41, 42]. Since the cutoff values for various body composition and systemic inflammatory parameters can vary significantly based on ethnicity and region, there is no universally accepted consensus on these cutoff values. We used X‐tile software and considered sex‐based differences to establish the cutoff values for body composition parameters based on our own cohort [21]. The computed cutoff values for NLR, PLR, LMR, SMI, MA, SATI, VSR, and SMR showed favorable ability to differentiate the patients’ OS outcomes and were consistent with those reported in previous Asian studies [38, 39, 43]. Our study expands on individual indicators mentioned by integrating them into a single PNSS and demonstrates that the PNSS can effectively predict the impact of p‐HIPEC on patient survival. Similar studies have shown that combining multimodal biomarkers enhances prognostic accuracy in cancer patients [44, 45]. The calibration plots for 3‐ and 5‐year OS rates demonstrated a good alignment with the ideal model, affirming its reliability as a prognostic tool. The classic pathological TNM staging system from the American Joint Committee on Cancer lacks sufficient accuracy in stratifying patients within the same stage, as it does not address for tumor behavior and treatment history variations. In this study, a specific PNSS that integrated nutritional biomarkers demonstrated satisfactory accuracy compared to the TNM staging system, suggesting that PNSS could offer a complementary value to the TNM system, enhancing patient stratification and providing more personalized clinical guidance.

PNSS effectively stratified high‐ and low‐risk patients, with results validated in both the primary and validation cohorts of LAGC patients who underwent radical surgery and p‐HIPEC. This stratification helps preselecting patients likely to benefit from p‐HIPEC based on their nutritional status. For high‐risk patients with a potential for worse prognosis, more intensive nutritional support and closer monitoring during the perioperative period may be necessary for this subgroup. Future research is required to explore the link between body composition and systemic inflammation within the context of GC and HIPEC. The clinical trials investigating precision nutritional interventions for high‐risk groups constitute the focus of the authors’ current research endeavors.

However, this study has some limitations. First, the retrospective and single‐center nature of our study may introduce selection bias and limit the generalizability of our findings. Future prospective, multicenter studies are needed to validate the findings and ensure the generalizability of the PNSS. Additionally, while the PNSS was effective in predicting OS in GC patients undergoing curative surgery with p‐HIPEC, its role in predicting other outcomes, such as disease recurrence and PM, remains to be explored.

## 5. Conclusion

In conclusion, systemic inflammatory response is linked to adverse body composition conditions in GC patients treated with curative surgery and p‐HIPEC. The PNSS integrates body composition parameters and inflammatory–nutritional indices; the model provides an accurate and comprehensive approach to risk stratification, with the potential to guide personalized treatment strategies and improve patient outcomes.

**Nomenclature**
AGR: albumin‐to‐globulin ratio
ALB: albumin
ASA: American Society of Anesthesiologists
CIs: 95% confidence intervals
CRP: C‐reactive protein
CT: computed tomography
GC: gastric cancer
GLO: globulin
HIPEC: hyperthermic intraperitoneal chemotherapy
HU: Hounsfield unit
K‐M: Kaplan–Meier
LAGC: locally advanced gastric cancer
MA: muscle attenuation
MUST: Malnutrition Universal Screening Tool
NLR: neutrophil‐to‐lymphocyte ratio
NRS‐2002: Nutritional Risk Screening 2002
ORs: odds ratios
OS: overall survival
PAR: platelet‐to‐albumin ratio
p‐HIPEC: prophylactic hyperthermic intraperitoneal chemotherapy
PLR: platelet‐to‐lymphocyte ratio
PMs: peritoneal metastases
PNI: prognostic nutritional index
PNSS: prognostic nutritional scoring system
PONS: Patient‐Generated Subjective Global Assessment–Short Form
ROC: receiver‐operating characteristic
RR: relative risk
SAT: subcutaneous adipose tissue
SATI: subcutaneous adipose tissue index
SII: systemic immune‐inflammation index
SIRS: systemic inflammatory response syndrome
SM: skeletal muscle
SMI: skeletal muscle index
SMR: skeletal muscle refined by muscle attenuation
VAT: visceral adipose tissue
VSR: VAT to SAT ratio
WBC: white blood cell count

## Author Contributions

Ruiqing Liu, Yahya Ahmed, and Duo Li contributed to the study conception and design. Data collection and data analysis were performed by Ruiqing Liu, Zhibo Wang, Jingnong Liu, and Yuandi Wang. The original draft was written by Ruiqing Liu, Dongsheng Wang, and Duo Li.

## Funding

This work was financially supported by the China Postdoctoral Science Foundation (No. 2023M741858), Beijing Xisike Clinical Oncology Research Foundation (Y‐NESTLE2022QN‐0230), and China Crohn’s & Colitis Foundation (CCCF) (CCCF‐QF‐2023C18‐3).

## Disclosure

All authors commented on the manuscript and read and approved the final version.

## Ethics Statement

The studies involving humans were approved by the Affiliated Hospital of Qingdao University (QYFYWZLL28673). The studies were conducted in accordance with the local legislation and institutional requirements. Written informed consent for participation was not required from the participants or the participants’ legal guardians/next of kin in accordance with the national legislation and institutional requirements.

## Conflicts of Interest

The authors declare no conflicts of interest.

## Supporting information


**Supporting Information** Additional supporting information can be found online in the Supporting Information section. Table S1: Determination of cutoff values for inflammation‐nutritional and body composition parameters. Table S2: Correlations between inflammation‐nutritional markers and body composition‐based markers (primary cohort and validation cohort). Table S3: Univariate and multivariate Cox analysis in validation cohort among clinicopathologic parameters, systemic inflammatory markers, and poorer body composition conditions Table S4: Time‐dependent predictive accuracy of three models: T‐N stage, inflammatory markers, and body compositions. Table S5: Comparison of overall survival (OS) outcomes between different groups stratified by nomo scores. Figure S1: Survival curves for inflammatory–nutritional variables with cutoff values determined by X‐tile analysis.

## Data Availability

The data that support the findings of this study are available from the corresponding author upon reasonable request.
