# Supplementary material for: A Prognostic Nutritional Scoring System Integrating Inflammatory Markers and Body Composition in Gastric Cancer Patients Undergoing Curative Resection With Prophylactic HIPEC
Source: Gastroenterol Res Pract. 2026 May 14;2026:7959410. doi: 10.1155/grp/7959410 (PMC13173548; doi:10.1155/grp/7959410)

**Supplementary materials**

**Table S1** Determination of cutoff values for inflammation-nutritional and body composition parameters

| **Cut-off values determined by X-tile** | | | | | |
| --- | --- | --- | --- | --- | --- |
|  | Cut-off values | P value | Chi Square value | RR | Definitions |
| NEU | 2.4 | 1.0000 | 1.7332 | 1.00/1.43 | Neutrophil count (*10^9^/L) |
| PLT | 325.0 | 1.0000 | 2.1470 | 1.00/1.27 | Platelet count (*10^9^/L) |
| LYM | 2.6 | 0.6786 | 2.8686 | 1.37/1.00 | Lymphocyte count (*10^9^/L) |
| ALB | 35.8 | 0.3288 | 4.9275 | 1.36/1.00 | Albumin (g/L) |
| MON | 0.3 | 1.0000 | 2.2954 | 1.00/1.95 | Monocyte count (*10^9^/L) |
| HB | 105.0 | 0.2195 | 5.9457 | 1.37/1.00 | Hemoglobin (g/L) |
| WBC | 5.5 | 1.0000 | 2.1582 | 1.19/1.00 | White cell count (*10^9^/L) |
| CRP | 3.2 | 0.0142 | 12.0614 | 1.00/1.57 | C-reactive protein (mg/L) |
| GLO | 22.4 | 1.0000 | 1.7755 | 1.00/1.52 | Globulin (g/L) |
| NLR | 1.9 | 0.021 | 5.9384 | 1.00/1.36 | Neutrophil count (*10^9^/L) / lymphocyte count (*10^9^/L) |
| PLR | 128.7 | <0.001 | 5.1477 | 1.00/1.34 | Platelet count *10^9^ /L) / lymphocyte count (*10^9^ /L) |
| PAR | 5.0 | 0.16 | 2.5581 | 1.00/1.58 | Platelet count(*10^9^/L) / Albumin(g/L) |
| PNI | 49.5 | 0.11 | 3.6660 | 1.30/1.00 | Albumin+5*total lymphocyte count (*10^9^ /L) |
| SII | 423.4 | 0.0041 | 9.1937 | 1.00/1.60 | Platelet count (*10^9^/L) * neutrophil count (*10^9^  /L) / lymphocyte count (*10^9^/L) |
| LMR | 4.7 | 0.0085 | 4.4174 | 1.37/1.00 | Lymphocyte count (*10^9^ /L) / monocyte count (*10^9^ /L) |
| AGR | 1.2 | 0.5950 | 3.3166 | 1.00/1.74 | Albumin / globulin |
| SM | 122.6  Male (116.7)  Female (132.5) | 0.0423 | 9.6887 | 1.38/1.00 | L3 skeletal muscle cross-sectional areas (cm2) |
| VAT | 76.2 | 0.45 | 2.2898 | 1.17/1.00 | Visceral adipose tissue cross-sectional areas (cm2) |
| SAT | 130.8 | 0.08 | 4.3993 | 1.33/1.00 | Subcutaneous adipose tissue cross-sectional areas (cm2) |
| MA | 44.6  Male (45.9)  Female (38.2) | 0.0044 | 9.9951 | 1.00/1.53 | Mean muscle attenuation (HU) computed from the same region that defined SM |
| SMI | 40.1  Male (42.3)  Female (35.5) | 0.0034 | 12.6328 | 1.42/1.00 | L3 skeletal muscle index: SM normalized for height (m2) |
| VSR | 1.6  Male (1.69)  Female (1.37) | 0.035 | 2.1702 | 1.00/1.60 | VAT to SAT ratio |
| VATI | 24.0 | 0.13 | 2.7371 | 1.16/1.00 | Visceral adipose tissue index: VAT normalized for height (m2) |
| SATI | 47.5 | 0.022 | 4.4826 | 1.28/1.00 | Subcutaneous adipose tissue index: SAT normalized for height (m2) |
| SMR | 2.9 | <0.0001 | 21.7941 | 1.75/1.00 | SM refined by MA: SM * MA |
| MAI | 10.6 | 0.007 | 0.8703 | 1.09/1.00 | Mean muscle attenuation index: MA normalized for height (m^2^) |
| **Cut-off values referring to previous studies** | | | | | |
| Sarcopenia | SMI: Women 34.90  Men 40.80 | NA | NA | NA | L3 skeletal muscle cross-sectional areas (cm^2^) normalized for height (m^2^) [1] |
| Myosteatosis | MA: Women 26.20  Men 34.50 | NA | NA | NA | Mean muscle attenuation (HU) computed from the same region that defined sarcopenia |
| Visceral obesity | VSR: Women 0.60  Men 0.70 | NA | NA | NA | Ratio of visceral-to-subcutaneous adipose tissue area (cm^2^) normalized for height (m^2^) |
| Sarcopenia obesity | NA | NA | NA | NA | The combination of sarcopenia and visceral obesity with the above definition |

***Footnotes.*** Cutoff values were determined using X-tile software for the specific inflammatory markers or body compositions in this cohort. The definition of sarcopenia, myosteatosis, visceral obesity, and sarcopenia obesity otherwise were adopted from established previous study [1]."

1. Xiong J, Wu Y, Hu H, et al. Prognostic Significance of Preoperative Sarcopenia in Patients with Gastric Cancer Liver Metastases Receiving Hepatectomy. Front Nutr. 2022; 9:878791. Published 2022 May 10. doi:10.3389/fnut.2022.878791

**Abbreviation:** NEU= Neutrophils. PLT=Platelets. LYM=Lymphocytes. ALB=Albumin. MON=Monocytes. HB=Hemoglobin. WBC=White Blood Cells. CRP=C-Reactive Protein. GLO=Globulins. NLR=Neutrophil-to-Lymphocyte Ratio. PLR=Platelet-to-Lymphocyte Ratio. PAR= Platelet-to-Albumin Ratio. PNI=Prognostic Nutritional Index. SII Systemic Immune-inflammation Index=LMR Lymphocyte-to-Monocyte Ratio. AGR=Albumin-to-Globulin Ratio. SM=Skeletal Muscle area. VAT=Visceral Adipose Tissue area. SAT=Subcutaneous Adipose Tissue area. MA=Muscle Attenuation (a measure of muscle density/fat content). SMI=Skeletal Muscle Index (SM normalized for height). VSR= Visceral-to-Subcutaneous fat Ratio. VAT=Visceral Adipose Tissue Index (VAT normalized for height). SATI=Subcutaneous Adipose Tissue Index (SAT normalized for height). SMR=Sarcopenia-Myosteatosis Risk score. MAI=Muscle Attenuation Index.

**Table S2** Correlations between inflammation-nutritional markers and body composition-based markers (primary cohort and validation cohort)

Primary cohort:


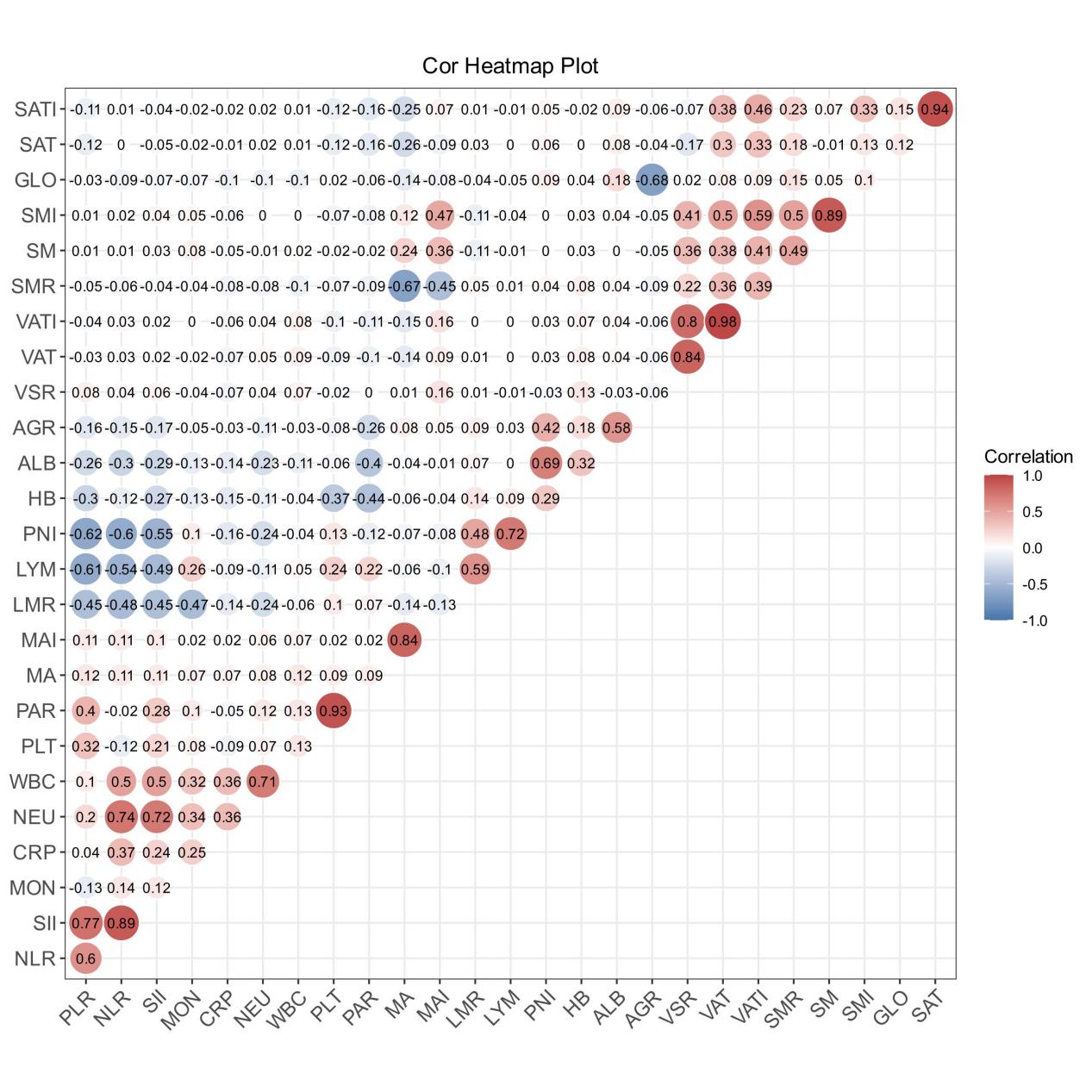


Validation cohort:

**
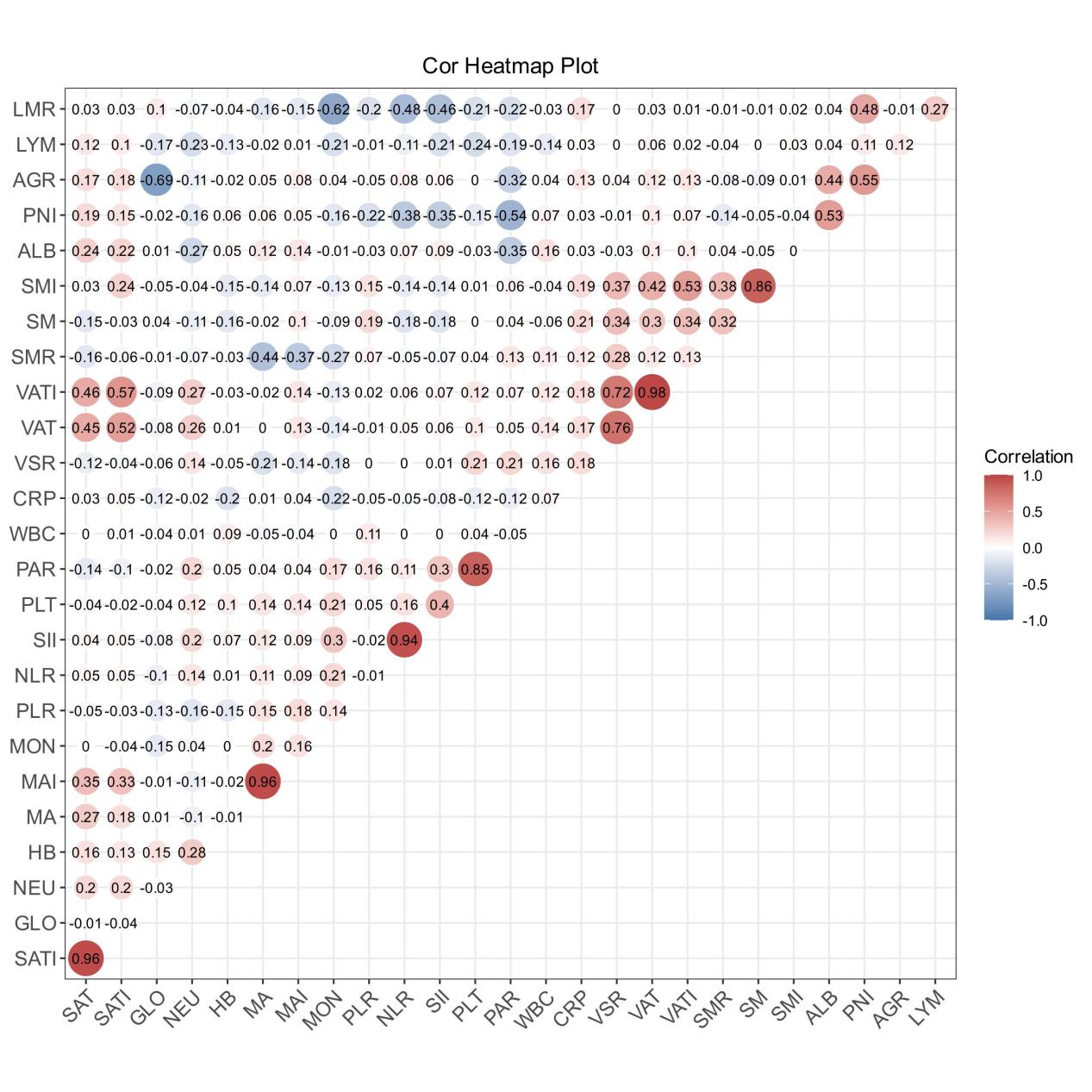
**

**Table S3** Univariate and multivariate Cox analysis in validation cohort among clinicopathologic parameters, systemic inflammatory markers, and poorer body composition conditions

|  | Univariable | | | Multivariable | | |
| --- | --- | --- | --- | --- | --- | --- |
|  | HR | 95%CI | P-value | HR | 95%CI | P-value |
| Age | 0.639 | 0.31-1.34 | 0.234 |  |  |  |
| Gender | 0.73 | 0.36-1.49 | 0.389 |  |  |  |
| T stage | 3.34 | 1.28-8.76 | 0.014 | 1.79 | 0.57-5.58 | 0.316 |
| N stage | 3.221 | 1.47-7.06 | 0.004 | 1.307 | 0.47-3.62 | 0.606 |
| NRS2002 (>2) | 1.234 | 0.57-2.70 | 0.598 |  |  |  |
| Histology | 1.595 | 0.65-3.91 | 0.308 |  |  |  |
| Tumor size | 1.289 | 0.63-2.64 | 0.488 |  |  |  |
| Differentiation degree | 1.598 | 0.65-3.92 | 0.307 |  |  |  |
| Vascular invasion | 0.929 | 0.44-1.94 | 0.844 |  |  |  |
| Neoadjuvant therapy | 1.396 | 0.49-4.01 | 0.535 |  |  |  |
| NLR (> 1.9) | 2.681 | 1.20-6.00 | 0.016 | 1.627 | 0.57-4.68 | 0.367 |
| PLR (> 128.7) | 3.541 | 1.45-8.65 | 0.005 | 3.72 | 1.17-11.78 | 0.026 |
| PAR (< 5.0) | 0.485 | 0.15-1.61 | 0.236 |  |  |  |
| PNI (< 49.5) | 1.24 | 0.47-3.30 | 0.663 |  |  |  |
| SII (< 423.4) | 0.872 | 0.37-2.04 | 0.752 |  |  |  |
| LMR (> 4.7) | 0.203 | 0.048-0.86 | 0.03 | 1.528 | 0.67-3.48 | 0.312 |
| SMI (< 40.1) | 0.31 | 0.14-0.66 | 0.003 | 0.427 | 0.18-1.01 | 0.052 |
| MA (< 44.6) | 2.15 | 1.05-4.39 | 0.036 | 1.376 | 0.59-3.19 | 0.456 |
| VSR (< 1.6) | 2.755 | 1.30-5.90 | 0.008 | 1.94 | 0.86-4.41 | 0.113 |
| VATI (< 24.0) | 0.741 | 0.34-1.62 | 0.454 |  |  |  |
| SATI (> 47.5) | 1.087 | 0.52-2.29 | 0.827 |  |  |  |
| SMR (< 2.9) | 2.755 | 1.30-5.85 | 0.008 | 2.746 | 1.22-6.17 | 0.014 |
| MAI (< 10.6) | 1.546 | 0.69-3.48 | 0.292 |  |  |  |

**Abbreviation:** HR=Hazard Ratio. IQR=Interquartile Range. CI=Confidence Interval. BMI=Body Mass Index, NRS2002=Nutritional Risk Screening 2002. NLR=Neutrophil-to-Lymphocyte Ratio. PLR=Platelet-to-Lymphocyte Ratio. PAR= Platelet-to-Albumin Ratio. PNI=Prognostic Nutritional Index. SII Systemic Immune-inflammation Index=LMR Lymphocyte-to-Monocyte Ratio. AGR=Albumin-to-Globulin Ratio. SM=Skeletal Muscle area. VAT=Visceral Adipose Tissue area. SAT=Subcutaneous Adipose Tissue area. MA=Muscle Attenuation (a measure of muscle density/fat content). SMI=Skeletal Muscle Index (SM normalized for height). VSR= Visceral-to-Subcutaneous fat Ratio. VAT=Visceral Adipose Tissue Index (VAT normalized for height). SATI=Subcutaneous Adipose Tissue Index (SAT normalized for height). SMR=Sarcopenia-Myosteatosis Risk score. MAI=Muscle Attenuation Index.

**Table S4** Time dependent predictive accuracy of three models: T-N stage, inflammatory markers and body compositions.

**Primary cohorts:**

| Month | TNM | Inflammatory_nutritional_markers | Body_composition | Nomogram |
| --- | --- | --- | --- | --- |
| 9 | 0.627095 | 0.7681564 | 0.4511173 | 0.6662011 |
| 12 | 0.4585687 | 0.7683616 | 0.3879473 | 0.6280603 |
| 15 | 0.6194581 | 0.6958128 | 0.4622332 | 0.6211002 |
| 18 | 0.5907917 | 0.6039157 | 0.4625645 | 0.5378657 |
| 21 | 0.5959737 | 0.6349945 | 0.5210837 | 0.6077436 |
| 24 | 0.6559061 | 0.6380959 | 0.6203112 | 0.6802697 |
| 27 | 0.7176063 | 0.6411286 | 0.6715164 | 0.7299172 |
| 30 | 0.7002910 | 0.6645138 | 0.7133047 | 0.7759755 |
| 33 | 0.6735894 | 0.6966293 | 0.6680988 | 0.7710680 |
| 36 | 0.7169611 | 0.6787557 | 0.6639333 | 0.7563786 |
| 39 | 0.6854919 | 0.6740118 | 0.6816325 | 0.7621915 |
| 42 | 0.6932538 | 0.6840485 | 0.6740909 | 0.7600161 |
| 45 | 0.6382882 | 0.6827666 | 0.7016297 | 0.7736090 |
| 48 | 0.6484138 | 0.6695666 | 0.6991129 | 0.7604013 |
| 51 | 0.6280571 | 0.6437077 | 0.6817267 | 0.7326322 |
| 54 | 0.6712284 | 0.5684953 | 0.7046998 | 0.6989988 |
| 57 | 0.7833778 | 0.5735449 | 0.7199000 | 0.7019909 |
| 60 | 0.7833778 | 0.6372379 | 0.6747175 | 0.7113844 |

**Validation cohorts:**

| Month | TNM | Inflammatory_nutritional_markers | Body_composition | Nomogram |
| --- | --- | --- | --- | --- |
| 9 | 0.8313253 | 0.497992 | 0.3674699 | 0.435743 |
| 12 | 0.75 | 0.55375 | 0.58375 | 0.60625 |
| 15 | 0.6834328 | 0.5560724 | 0.6927705 | 0.7046885 |
| 18 | 0.687667 | 0.5623681 | 0.6943363 | 0.710113 |
| 21 | 0.6633991 | 0.5943163 | 0.634393 | 0.7004608 |
| 24 | 0.6825586 | 0.512593 | 0.6271131 | 0.6479876 |
| 27 | 0.6981819 | 0.5055174 | 0.6534886 | 0.6695141 |
| 30 | 0.6990263 | 0.4823066 | 0.6838597 | 0.6690135 |
| 33 | 0.6872296 | 0.559301 | 0.6011274 | 0.6665010 |
| 36 | 0.6962868 | 0.5462533 | 0.6194312 | 0.6741109 |
| 39 | 0.7019415 | 0.6000494 | 0.6368208 | 0.7202854 |
| 42 | 0.6609678 | 0.5964534 | 0.6453381 | 0.7128367 |
| 45 | 0.6586805 | 0.5193235 | 0.6173259 | 0.6522242 |
| 48 | 0.595941 | 0.5017756 | 0.6136155 | 0.6415372 |
| 51 | 0.5778371 | 0.4967358 | 0.6078812 | 0.6417222 |
| 54 | 0.5208282 | 0.3419802 | 0.6071914 | 0.5248349 |
| 57 | 0.6438152 | 0.4874072 | 0.5502943 | 0.5757671 |
| 60 | 0.6404943 | 0.6143694 | 0.5542453 | 0.6858372 |

**Table S5** Comparison of overall survival (OS) outcomes between different groups stratified by nomo scores

|  | No. of patients | 3-year OS rate, (%) ± SE | 5-year OS rate, (%) ± SE | estimated median times to death, months | 95% CI | p value |
| --- | --- | --- | --- | --- | --- | --- |
| Primary cohort (n=181) |  |  |  |  |  | <0.001 |
| nomo_low | 69 | 82.6±4.6 | 62.7±8.1 | 61.193 | 55.617-66.769 |  |
| nomo_median | 77 | 58±5.7 | 30.4±6.4 | 40.07 | 34.583-45.557 |  |
| nomo_high | 35 | 27.7±7.8 | 0 | 27.2 | 25.363-29.037 |  |
| Validation cohort (n=86) |  |  |  |  |  | <0.001 |
| nomo_low | 35 | 82.9±6.4 | 54.3±14.4 | 63.93 | 45.202-58.879 |  |
| nomo_median | 38 | 70.4±7.5 | 66.9±7.9 | 59.213 | 51.408-67.018 |  |
| nomo_high | 13 | 38.5±13.5 | 0 | 24.73 | 21.339-34.719 |  |

**Note:** CI, confidence interval; p value was obtained from Log-rank analysis. The cut-off value of nomo-score for differentiating nomo-low and nomo-median is 0.6. The cut-off value of nomo-score for differentiating nomo-median and nomo-high is 1.17.

**Supplementary Figure S1** Survival curves for inflammatory nutritional variables with cutoff values determined by X-tile analysis.


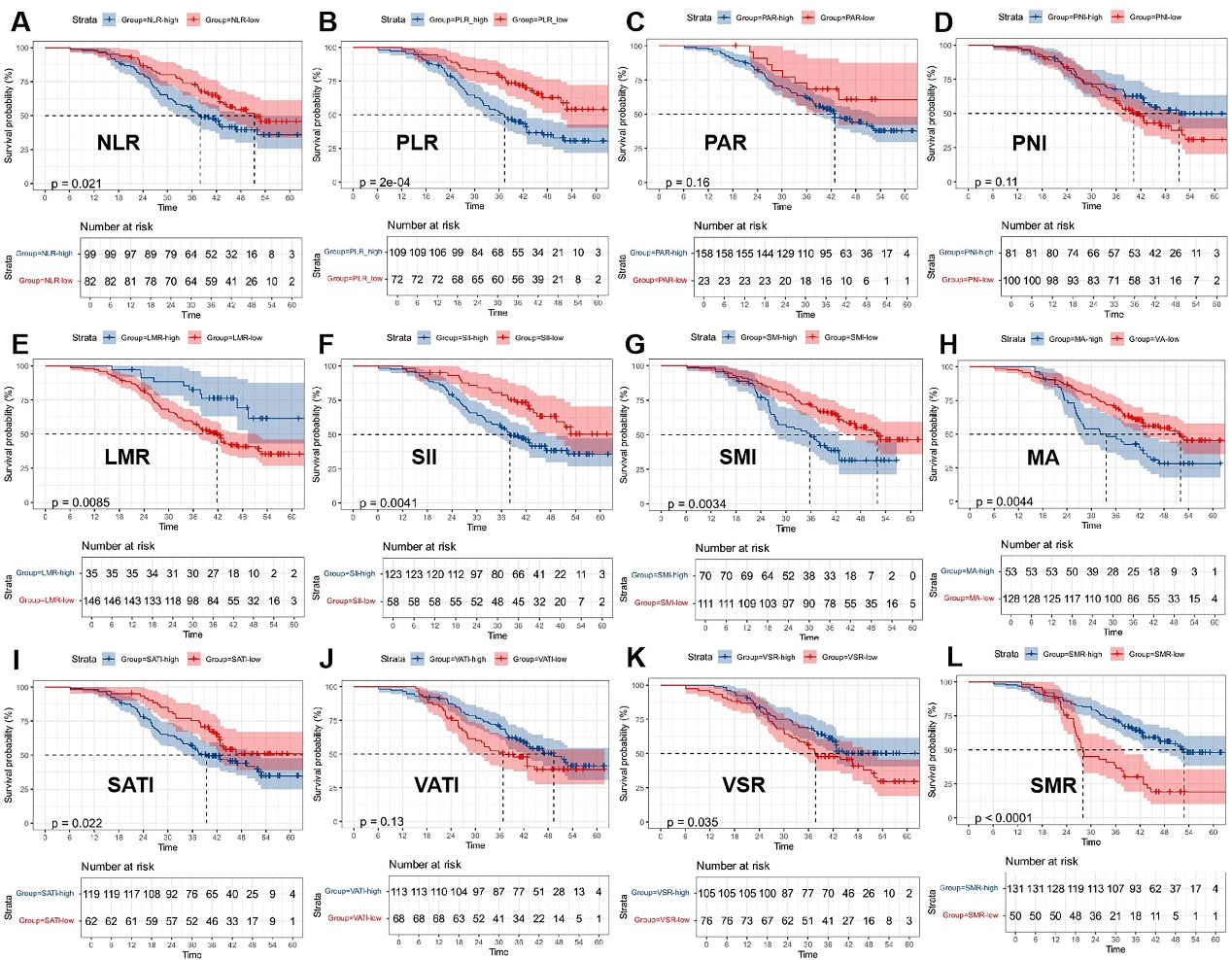

Supplement: Supplementary file 1 — Supporting Information Additional supporting information can be found online in the Supporting Information section. Table S1: Determination of cutoff values for inflammation‐nutritional and body composition parameters. Table S2: Correlations between inflammation‐nutritional markers and body composition‐based markers (primary cohort and validation cohort). Table S3: Univariate and multivariate Cox analysis in validation cohort among clinicopathologic parameters, systemic inflammatory markers, and poorer body composition conditions Table S4: Time‐dependent predictive accuracy of three models: T‐N stage, inflammatory markers, and body compositions. Table S5: Comparison of overall survival (OS) outcomes between different groups stratified by nomo scores. Figure S1: Survival curves for inflammatory–nutritional variables with cutoff values determined by X‐tile analysis. [file GRP-2026-7959410-s001.docx]
